# ESBL-Positive Enterobacteria Isolates in Drinking Water

**DOI:** 10.3201/eid1806.111214

**Published:** 2012-06

**Authors:** Hilde De Boeck, Berthe Miwanda, Octavie Lunguya-Metila, Jean-Jacques Muyembe-Tamfum, Ellen Stobberingh, Youri Glupczynski, Jan Jacobs

**Affiliations:** Institute of Tropical Medicine, Antwerp, Belgium (H. De Boeck, J. Jacobs);; Institut National de Recherche Biomédicale, Kinshasa, Democratic Republic of the Congo (B. Miwanda, O. Lunguya-Metila, J.J. Muyembe-Tamfum);; Maastricht University Medical Center, Maastricht, the Netherlands (E. Stobberingh);; Cliniques Universitaires Université Catholique de Louvain de Mont-Godinne, Yvoir, Belgium (Y. Glupczynski)

**Keywords:** Enterobacteriaceae, enterobacteria, CTX-M enzymes, extended-spectrum β-lactamase, ESBL, Democratic Republic of the Congo, water quality, sachet packaged water, bacteria, drinking water

**To the Editor:** Extended-spectrum β-lactamase (ESBL)–producing members of the family *Enterobacteriaceae* (enterobacteria) are a worldwide problem (*1*), but little data are available from central Africa (*2*). In recent years, ESBL-producing *Enterobacteriaceae* isolates have shifted from the hospital to the community and the environment (*1*). The aim of this study was to assess the presence of ESBL-producing *Enterobacteriaceae* isolates in sachet-packaged water bags sold as drinking water in the streets of Kinshasa, the capital of Democratic Republic of the Congo.

In November 2009 and June 2010, a total of 101 sachet-packaged water bags were bought from street vendors in 9 of 24 municipalities (covering residential areas and slums) of the city of Kinshasa. The bags were transported in ice coolers and processed within 4 hours of collection. We filtered 100 mL of each sample through 0.45-µm pore size filters (Sartorius, Goettingen, Germany). The filters were then transferred to an agar plate containing mEndoLES agar (Difco, Franklin Lakes, NJ, USA) and incubated at 35°C for 24 hours.

Growing colonies were subcultured on Kligler iron agar (Oxoid, Cambridge, UK), and gram-negative glucose-fermenting isolates were identified to the species level and assessed for antimicrobial drug susceptibility with Microscan NBC42 panels (Siemens Healthcare Diagnostics Inc., West Sacramento, CA, USA). Isolates labeled by Microscan as ESBL producers were confirmed by the double-disk method, which compared 1 disk containing cefotaxime with 1 disk containing cefotaxime and clavulanic acid and 1 disk containing ceftazidime with 1 disk containing ceftazidime and clavulanic acid (Rosco Diagnostica, Taastrup, Denmark), according to the Clinical and Laboratory Standards Institute guidelines (*3*). We used *Escherichia coli* ATCC 25922 and *Klebsiella pneumoniae* ATCC 700603 as control strains. Detection and identification of ESBL-producing *bla* genes were carried out by using a commercial multiplex ligation PCR microarray CT 101 (Check-Points Health BV, Wageningen, the Netherlands) (*4*).

A total of 101 sachet-packaged water bags were purchased at random from different vendors: 88 sealed and branded bags (of 68 different brands) and 13 hand-tied unbranded bags. The precise origin of the water could not be determined because vendors are unlicensed resellers and most producers are not registered. Water bags lacked essential information such as contact addresses, batch number, and production and expiration dates. Because we also observed empty branded sheets of bags for sale on the market, we assumed that many of the branded bags are simply filled with tap water or water from other supplies without any prior treatment. None of the water bags tested for chlorine (36 of 101) contained free chlorine levels >0.1 mg/L. Nearly one third of the water bags were contaminated with *Enterobacteriaceae* isolates (22/88 branded bags and 9/13 hand-tied bags). The bags were obtained in townships and residential quarters.

Overall, 150 nonduplicate *Enterobacteriaceae* isolates were recovered. The main species were *K. pneumoniae* (56.0% of isolates found in 23/101 of water bags) and *Enterobacter* spp. (30.6%, in 20/101 water bags); *Citrobacter* spp. accounted for 4.7% of isolates and *E. coli* for 3.3%. Eight isolates (5.3 %) were confirmed as ESBL producers by antimicrobial drug susceptibility tests, and they were recovered from 2 branded and 2 hand-tied bags. The species, microarray results, and the associated drug resistance are listed in the Table. Five isolates carried *bla*_CTX-M_ genes belonging to CTX-M1 group, and 3 isolates carried *bla*_SHV_ variants. No TEM-ESBL genes were detected. On the basis of checkpoint results and previously validated data, we further categorized the SHV G238S mutation as SHV-2-like and the double SHV G238A + SHV E240K mutation as SHV-18 (*5*).

**Table:** ESBL-producing *Enterobacteriaceae* recovered from sachet-packaged water bags in Kinshasa, Democratic Republic of the Congo*

Isolate no.	Species	Microarray CT101 result	Associated resistance†		Folate PI
			Aminoglycosides	Fluoroquinolones	
44	*Citrobacter freundii*	CTX-M1 group	AMK, GEN, TOB	CIP, LEV, MXF, NXN	T/S
48	*Citrobacter freundii*	CTX-M1 group	AMK, GEN, TOB	CIP, LEV, MXF, NXN	T/S
152	*Enterobacter cloacae*	SHV G238S (SHV-2 like)	NA	NA	T/S
154	*Klebsiella pneumoniae*	CTX-M1 group	GEN, TOB	CIP, MXF	T/S
163	*Citrobacter freundii*	CTX-M1 group	NA	MXF	T/S
165	*Klebsiella pneumoniae*	CTX-M1 group	GEN, TOB	NA	T/S
170	*Klebsiella pneumoniae*	SHV G238A+E240K(SHV-18)	GEN, TOB	MXF	NA
171	*Escherichia coli*	SHV G238A+E240K(SHV-18)	GEN, TOB	NA	NA

*ESBL, extended-spectrum β-lactamase; PI, pathway inhibitors; AMK, amikacin; GEN, gentamicin; TOB, tobramycin; CIP, ciprofloxacin; LEV, levofloxacin; MXF, moxifloxacin; NXN, norfloxacin; T/S, trimethoprim/sulfamethoxazole; NA, not applicable. †Based on interpretive breakpoints as indicated in Clinical and Laboratory Standards Institute guidelines M100-S18, published January 2008.

ESBL-producing *Enterobacteriaceae* isolates constitute a major public health concern in industrialized and resource-poor settings. Few reports are available from Africa, although hospital-associated ESBL producers have been described in Cameroon and the Central African Republic (*6**,**7*). ESBL-producing bacteria have been recovered from different sources in the community, including food and companion animals (*8**,**9*), and 1 recent study from India reported that a substantial number of tap water samples were contaminated with carbapenemase *bla*_NDM-1_ producing organisms (*10*).

Kinshasa is the second-largest city in sub-Saharan Africa. In 2008, of its estimated 8.7 million inhabitants, only 46%had access to safe drinking water, and 23% had access to improved sanitation facilities according to the World Bank. Opportunistic pathogens in drinking water and poor sanitary conditions may increase the risk of developing infectious enterocolitis for consumers, especially for those who are immunocompromised. It can eventually lead to chronic intestinal carriage of multidrug-resistant organisms. The presence of ESBL producers in the intestinal flora could also lead to horizontal transfer of drug resistance genes from commensal flora to enteric pathogens. This emergence of ESBL-producing bacteria and further community-associated infections poses a public threat, especially in low-resource countries where surveillance is suboptimal and empiric treatment of invasive infections often includes third-generation cephalosporins.
